# Immune Regulatory Genes Are Major Genetic Factors to Behcet Disease: Systematic Review

**DOI:** 10.2174/1874312901812010070

**Published:** 2018-06-29

**Authors:** Yan Deng, Weifeng Zhu, Xiaodong Zhou

**Affiliations:** 1 The Second Affiliated Hospital of Nanchang University, Nanchangine>, China; 2 Department of Internal Medicine/Rheumatology, University of Texas Health Science Center at Houston McGovern Medical School, Houston, USA; 3 College of Basic Medical Sciences, Nanchang University, Nanchang, China

**Keywords:** Behcet's disease, genetic association, HLA, MICA, CIITA, ERAPI

## Abstract

Behcet's disease (BD) is a chronic refractory multi-system autoimmune disorder that occurs in a genetically susceptible host. Multiple genetic factors have been identified that may contribute to the pathogenesis of BD. The major genes with polymorphisms associated with BD include HLA-B and -A, CIITA, ERAP1, MICA, IL10, IL12A, IL12RB2, IL23R, MEFV, IRF8, TNFAIP3, REL, TLR4, NOD1,2, CCR1,CCR3, GIMAP1,2,4, KLRC4, STAT4, NCOA5, FOXP3, PSORS1C1, FUT2, UBAC2, SUMO4, ADO-EGR2, CEBPB-PTPN1, and JPKL-CNTN5. These genes encode proteins involved mainly in immune regulation and inflammation, and some in transcription and post-translational modification. A complete view of these BD-associated genes may provide a clue to this complex disease in terms of its pathogenesis and exploring potentially targeted therapies for BD.

## 1 INTRODUCTION

Behcet's Disease (BD) is a chronic inflammatory disorder, characterized by refractory genital and oral ophthous ulcers, uveitis, skin lesions, and manifestations in other systems including gastrointestinal tract, central nervous system, vascular system, joints, kidneys, and lungs [1]. BD was found primarily in populations along the ancient silk Route from the Mediterranean Basin across Asia to Japan [2]. The prevalence of BD was reported to vary widely in Western (0.12-7.5 per 100,000) [3] and Eastern countries (6.3-14 per 100,000) [4], which may be due to ethnic disparities, and risk factors. The highest incidence was reported in Turkey from 80 to 420 per 100,000 population [5-7]. Positive history of BD in families is 31.2% [8], which indicates genetic contribution to the disease by comparing to general population. The male-to-female ratio was reported from 1.22 to 1 [9]. An association was found between the male gender and vascular lesions [10]. The first reported genetic association of BD was found in the Human Leukocyte Antigen (HLA) region in the Japanese population [11]. HLA-B51 appears to be the most strongly associated known genetic risk to BD [12]. However, it accounts for less than 20% of the overall genetic risk, which suggests that other genetic factors are also important. Here we review genetic studies of BD reported from 1973 to January 2018. A complete list of BD-associated genes and genetic loci are summarized in Table **1**.

A comprehensive search was conducted on PubMed, Embase, Web of Science, and the HuGE Navigator databases. The PubMed and Embase database were searched by a combination of MeSH, Emtree headings and text words. Exclusion criteria included case reports, studies with duplicate data, and non-English language papers.

## 2 HLA AND HLA-RELATED GENES ARE STRONGLY ASSOCIATED WITH BD

### 2.1 HLA

Human Leucocyte Antigen (HLA) genes located on chromosome 6. HLA genes were among the most polymorphic genes in the human genome. To which several theories have been proposed to explain, including overdominance (heterozygote superiority) [13], negative frequency dependent selection (rare allele advantage) [14], and selection varying in time and space [15]. HLA genes were associated with susceptibility to almost all autoimmune diseases. Multiple HLA alleles have been associated with BD. Among them, HLA-B51 is considered the strongest genetic risk factor for BD, which has been verified in multiple studies and in different ethnic populations [16-26]. The population attributable risk (PAR) of HLA-B5/B51 was estimated to be 52.2% for BD patients in Southern Europe, 49.9% in Middle East/North Africa, 44.4% in East Asia, and 31.7% in Northern Europe [27]. Other HLA alleles including BD-risk HLA-A02, -A24, -A26, -A31, -B27, -B57, and BD-protective HLA-A03, -B15, -B35, -B49, -B58 were also reported in different populations [26, 28-33].

In addition to susceptibility, HLA-A26:01 was also associated with poor visual prognosis in Japanese BD patients with uveitis [34], and with a high prevalence of posterior uveitis in Korean BD patients [35]. HLA-A26:01, -A02:07 and -A30:04 were associated with skin lesions and arthritis, uveitis, vascular lesions, genital ulcers, and a positive pathergy test in the Korean and Japanese populations [36].

These findings suggest that HLA alleles reflect clinical manifestations and prognosis, indicating the possibility of a clinical use as a biomarker for diagnostic or prognostic classification of BD patients.

### 2.2 CIITA

The class II major histocompatibility complex transactivator (CIITA) acts as a transcriptional coactivator that regulates the MHC class II genes, IL-4, IL-10 and other immune-mediating genes [37], which has been implicated in various inflammatory and autoimmune diseases [37, 38]. A recent study of a Chinese Han population indicated that CIITA SNP rs12932187 G allele and GG genotype were risk factors to BD, and the GG carriers had a higher expression of the CIITA gene and a lower level of IL-10 protein secretion from the peripheral blood monocytes (PBMC) in response to Lipopolysaccharide (LPS) stimulation [39].

### 2.3 ERAP1

Endoplasmic Reticulum Aminopeptidase 1 (ERAP1) functions to trim peptides in the endoplasmic reticulum to optimize their length for MHC-I binding. The SNPs rs10050860 and rs17482078 of the ERAP1 gene encoding p.Asp575Asn and Arg725Gln, respectively, were found to recessively confer risk to BD in Turkish population [40]. The ERAP-BD association was replicated in a Chinese cohort [41]. Further analysis of the Turkish and Spanish cohorts indicated that the peptidase variant ERAP1 p.Arg725Gln contributing to BD susceptibility may act through an interaction with the HLA-B*51 protein [40, 42]. Functional correlation studies indicated that the expression of ERAP1 in active BD patients was found significantly lower than that in healthy controls [41]. ERAP1 expression in AA carriers of SNP rs1065407 and CC carriers of SNP rs10050860 was higher than that observed in AC/CC carriers or CT/TT carriers by lipopolysaccharide (LPS)-stimulated peripheral blood mononuclear cell (PBMCs), respectively [41, 42].

### 2.4 MICA

The major histocompatibility complex class I chain related gene A (MICA) is a non-classic HLA gene [43]. It functions in immune activation under cellular stress conditions, such as infections, tissue injury, pro-inflammatory signals, and malignant transformation. Similar to classic HLA genes, its DNA sequence is highly polymorphic [44]. According to IMGT/HLA database [https://www.ebi.ac.uk/ipd/imgt/hla/align.html], there are more than 100 identified MICA alleles. In addition, the codon 295 located in the Transmembrane (TM) region has a tri-nucleotide microsatellite polymorphism (GCT)n that is designated as An (A4, A5, A6, A9) allele, and a five repetition of GCT may coexist with a guanosine insertion that is designated as A5.1 [45].

The incidence of BD was strongly associated with MICA*009, *019 allele in a Spanish population [46], and MICA-A6 allele in Japanese patients [47, 48]. The association between MICA-A6 and BD appeared to be independent from potential Linkage Disequilibrium (LD) effect of HLA-B51 in a Korean population study in which they examined the HLA-B51-negative BD patients [49].

On the other hand, MICA-A5.1 demonstrated a negative correlation with ocular lesions and iridocyclitis in BD patients [49, 50]. MICA-A9 was associated with BD patients who had less severe complications including uveitis, thrombosis, and neurological and intestinal involvement [51].

## 3 MULTIPLE INTERLEUKINS (IL) FAMILY GENES ARE INVOLVED IN SUSCEPTIBILITY TO BD

### 3.1 IL-10

IL-10 protein is an anti-inflammatory cytokine that inhibits the costimulatory activity of macrophages for T cell and NK cell activation and, production of proinflammatory cytokines such as IL-1, IL-6, IL-12, tumor necrosis factor (TNF), and interferon gamma (IFN-γ) [52, 53]. SNP rs1800871 located at the IL-10 promoter region was first reported to be associated with BD in the UK or Middle Eastern (ME) cohorts [54]. The Genome-Wide Association Studies (GWAS) revealed multiple BD-associated SNPs (rs1518111, rs1554286, rs1800871 and rs1800872) of the IL-10 in a Chinese cohort [55] and Turkey, Japanese, Korean [56, 57].

### 3.2 IL-12

IL-12 is composed of a 35 kD (IL-12p35 encoded by IL-12A) and a 40 kD (IL-12p40 encoded by IL-12B) subunits. It binds to a heterodimeric IL-12 receptor (IL-12R) that consists of IL-12Rβ1 (encoded by IL-12RB1and IL-12Rβ2 (encoded by IL-12RB2) [58]. The IL-12A and the IL-12RB2 genes were linked to BD [37, 52, 53]. IL-12A encodes IL-12p35 that plays a crucial role in activation of NK cells and polarization of the Th1 pathway through differentiation from naïve CD4+ T cells [59], which produce IFN-γ, IL-2, TNF-β and other cytokines [60]. A suggestive association of SNP rs1780546 of the IL-12A gene was reported in the Turkish and European BD cohorts [40, 61], and SNP rs17810458 was reported in an European BD cohort [61].

### 3.3 IL-23R and IL-12RB2

IL-23 is a member of the IL-12 family that share receptor and ligand chains with IL-12. However, it impacts on the development of Th17 cell responses that differs from IL-12 on Th1 [62]. The IL-23R and the IL-12RB1 genes encode for two subunits of the receptor for IL-23 [63] which are known to play a key role in neutrophil inflammation and in autoimmune diseases [64].

The GWAS identified the IL-23R SNP rs11209026 (Gly149Arg) in association with the Japanese cohort, and SNP rs76418789 (Arg381Gln) with the Turkish population [65]. In addition, the IL-23R/IL-12RB2 genes with multiple SNPs (rs1495965, rs924080 rs11209032, rs17375018, rs7517847, 10489629 and rs1966176) were associated with BD in the cohorts of Turkey [56], Japanese [57], Han Chinese [66, 67], Iranian [68], Western Algeria [69] and Korean [70].

## 4 GENES INVOLVED IN INFLAMMATION AND AUTOIMMUNITY ARE SIGNIFICANTLY ASSOCIATED WITH BD

### 4.1 MEFV

The Mediterranean fever (MEFV) protein or pyrin is an important regulator of innate immunity and the inflammatory response to IL-1β and IFN-γ [71]. MEFV SNPs rs61752717 (Met694Val), rs28940580(Met680Ile), rs3743930(Glu148Gln) have been linked to Familial Mediterranean fever [72] that share inflammatory nature and high prevalence in Middle Eastern and Mediterranean populations with BD. The MEFV gene polymorphisms Met694Val and Met680Ile were risk factors for BD and appeared consistent in multiple independent studies [65, 73-76]. In addition, specific BD risk polymorphisms (Met694Val, Met680Ile) of the MEFV gene were also associated with greater responsiveness to bacterial products [74, 75].

### 4.2 IRF8

Interferon Regulatory Factor (IRF) 8 is a transcription factor of the Interferon (IFN) Regulatory Factor (IRF) family that regulate expression of type I IFN stimulated genes, and play a vital role in regulating the development and function of a variety of immune cells [77]. Importantly, IRF8 can inhibit Th17 cell differentiation through its interaction with the Th17 master transcription factor, ROR-γt [78]. Two SNPs (rs17445836 and rs11642873) of the IRF8 gene were associated with BD in a Chinese cohort, and which appeared to regulate IRF8 expression and cytokine production [79]. Three other SNPs (rs11117433, rs142105922 and rs7203487) of the IRF8 gene were also associated with BD in the Turkish, Iranian and Japanese cohorts [80].

### 4.3 TNFAIP3

TNF-α–induced protein 3 (TNFAIP3) is a ubiquitin-modifying enzyme A20 that regulates inflammation through NF-κB signaling pathway, and it can be induced by TNF, toll like receptors (TLRs), IL-1R, and NOD2 signaling [81-84]. A genetic association between the TNFAIP3 gene SNPs (rs9494885, rs10499194 and rs7753873) and BD was reported in Han Chinese [85], but not in the European population [86]. A Japanese study of familial BD indicated that a missense mutation C243Y in A20/TNFAIP3 was likely responsible for increased production of human inflammatory cytokines by reduced suppression of NF-κB activation [87].

### 4.4 REL

The REL gene encodes for c-Rel, which is a member of the NF-κB family. Previous studies showed that Rel knockout mice do not develop autoimmune diseases [84, 85], which indicates its importance in the regulation of immune activity. The REL SNPs (rs842647) GG genotype and rs842647 G allele were reported to be risk factors to BD, and the latter was also associated with skin lesions in BD patients [88-90].

### 4.5 TLR2,4

Toll-Like Receptors (TLR) are transmembrane proteins that are implicated in pathogen recognition and activation of innate immunity [91]. The TLR2 SNP rs2289318 C allele and genotype CC and SNP rs3804099 CT genotype were significantly associated with ocular BD patients in a Chinese cohort. The TLR4 gene was associated with BD in multiple studies Japanese [92], Korea [93], Chinese [94] and Turkish populations, but not in Italian [95] and Tunisiam [96] cohorts. In addition, two BD protective *TLR*4 variants identified in Turkish cohort [70], p.Asp299Gly (rs4986790) and p.Thr399Ile (rs4986791) were associated with hyporesponsiveness to endotoxin [97].

### 4.6 NOD1-NOD2

Nucleotide-binding Oligomerization Domain (NOD) protein 1 and 2 are cytosolic protein that play important roles in initiating inflammation in response to microbial components such as those derived from bacterial peptidoglycan [98, 99]. Early studies suggested that Crohn’s disease-associated Arg702Trp (rs2066844) of the NOD2 gene, was protective from BD [100]. Later, other independent studies using both targeted resequencing and next generation sequencing approaches supported NOD2 variants were significantly associated with BD [65, 101]. Recently, NOD1 was also reported as a BD-associated gene, in which minor allele (G) of NOD1 SNP rs2075818 was significantly decreased in the patients of a Chinese cohort [39].

### 4.7 CCR1-CCR3

CCR1 and CCR3 proteins are two C-C motif chemokine Receptor (CCR) family members, which are composed of 7-transmembrane structures. They couple to G- proteins for signal transduction within cells and serve as key regulators of leukocyte trafficking and immune system homeostasis [102]. The CCR1-CCR3 locus SNP rs7616215 was first found to be associated with BD in the Turkish GWAS [40]. A later study of Chinese population indicated a strong association of three SNPs (rs13084057, rs13092160 and rs13075270) of the CCR1-CCR3 with BD [103]. The association of the CCR1 gene was also verified in Turkish, Japanese and Iranian cohorts [40, 104]. Further studies of CCR1 mRNA expression in primary human monocytes indicated that CCR1 expression and monocyte chemotaxis were reduced in individuals carrying the BD-risk allele [40].

### 4.8 GIMAP

GTPase of the Immunity-Associated Protein (GIMAP) family are expressed most extensively in the immune system and are differentially regulated during early human Th cell differentiation, especially in the course of Th1 differentiation [105]. It is also involved in survival and apoptosis of T cells and some other cell types [105, 106]. An early GWAS suggested a GIMAP cluster including SNPs in GIMAP1 (rs2286900), GIMAP2 (rs10266069, rs10256482) and GIMAP4 (rs1916012, rs1522596 and rs1608157) is a susceptibility locus for BD in Korean and Japanese populations [107]. However, the association was not replicated in later study of European cohort [108].

### 4.9 KLRC4

Killer cell lectin-like receptor subfamily C, member 4 (KLRC4) is a member of NKG2 receptor family that regulate NK cell function. A suggestive association between SNPs of KLRC1-4 and BD was found in the GWAS of Turkish and Japanese cohorts [40]. It was then verified in Iranian cohort [104].

## 5 GENES INVOLVED IN TRANSCRIPTION ACTIVATION OF IMMUNE REGULATION ARE ASSOCIATED WITH BD

### 5.1 STAT4

Signal transducer and activator of transcription-4 (STAT4) is a transcription factor that activates gene expression involved in functional regulation and differentiation of T-helper cells, natural killer (NK) cells, mast cells and dendritic cells [109]. It modulates differentiation of naïve T cells into Th1 and Th17 cells [60, 110, 111].

The association between the STAT4 gene and BD was first reported in a Han Chinese population [108]. It then was replicated in Korean, Turkish, Iranians [40, 104]. In addition, it was found that the risk allele A of STAT4 SNP rs897200 was associated with increased expression of the STAT4 gene, along with increased gene and protein expression of IL-17, which were correlated with a higher clinical severity score of BD patients [112].

### 5.2 NCOA5

Nuclear receptor coactivator-5 (NCOA5) protein regulates nuclear receptor subfamily 1 group D member 2 (NR1D*2*) and estrogen receptor 1 and 2 (ESR1 and ESR2) [113, 114]. NCOA5 SNP rs2903908 was associated with BD in the Finlang and the Turkish cohorts, and the CT genotype of rs2903908 was associated with genital ulceration and uveitis of the BD patients [115].

### 5.3 FOXP3

FOXP3 (forkhead box P3), also known as scurfin, is an important transcription factor regulating the development and function of Treg cells [116,117]. The SNP rs3761548 (3279 C/A) of the FOXP3 gene was significantly associated with BD in the North-Western Iranian population [118]. Another study showed that a low Copy Number Variant (CNV) of the FOXP3 gene conferred a risk to female BD patients, not male in a Chinese cohort [119].

## 6 OTHER GENES

### 6.1 PSORS1C1

Psoriasis susceptibility 1 candidate 1 (PSORS1C1) gene was initially recognized as it confers susceptibility to psoriasis [120] and psoriatic arthritis [121]. Recently, it was found to be a shared genetic factor in several other rheumatic diseases such as scleroderma [122], Crohn’s disease [123] and BD (SNP rs12525170) [124]. Although the function remains unclear, it is believed that PSORS1C1 contributes to the pathogenesis of autoimmunity [125, 126].

### 6.2 FUT2

The FUT2 gene encodes fucosyltransferase 2, which is involved in synthesis of the H antigen, the precursor of the ABO-histo-blood group antigen in body fluids and on the intestinal mucosa [127]. The association of FUT2 SNPs (rs632111, rs601338, rs602662, rs492602, rs681343 and rs281377) with BD was found in Iranian and Turkish population [128].

### 6.3 UBAC2

Ubiquitin-associated domain containing 2 (UBAC2) protein may be involved in protein localization in the endoplasmic reticulum [129]. Association of the UBAC2 gene with BD was first found in Turkish (SNP rs9513584) [130]. Additional BD-associated UBAC2 SNPs (rs7999348, rs9517723, rs3825427, rs9517668 and rs9517701) were found in Chinese, Italian and Japanese populations [131-133]. Moreover, the presence of BD-risk alleles of the rs9517723 was correlated with an increased expression of the UBAC2 gene which could induce over-activation of ubiquitination-related pathway leading to the development of ocular and CNS lesions in BD [133].

### 6.4 SUMO4

Small Ubiquitin-like Modifier 4 (SUMO4) is a member of the SUMO family that post-transcriptionally sumoylate proteins to regulate subcellular localization and/or enhance protein stability and activity [134]. It negatively regulated NFκB activity indicating its role in immune regulation [135]. The genetic association between the SUMO4 (SNPs rs237024, rs237026) and BD was first reported in a Chinese cohort, and that appeared independent from HLA-B51 [136]. The association was replicated in Tunisian and Korean cohorts [137,138], and specific polymorphisms were also associated with disease severity, skin lesions and vascular involvement of BD patients [137,138].

### 6.5 Loci at ADO-EGR2, CEBPB-PTPN1, JRKL-CNTN5

In addition to above genes, two genetic loci between the ADO and the EGR2 genes, and the CEBPB and the PTPN1 genes were associated with BD in a study of profiling immune-related loci in the Turkish, Iranian and Japanese cohorts [80]. The ADO encodes cysteamine (2-aminoethanethiol) dioxygenase that is involved in amino acid metabolism [139]. The EGR2 encodes early growth response protein 2 that is a transcription regulatory factor, and highly expressed in a population of migrating neural crest cells [140]. CEBPB encodes CCAAT/Enhancer Binding Protein Beta, a member of the CCAAT/enhancer binding protein family of basic leucine zipper transcription factors. It plays an important roles in controlling cell differentiation and proliferation, as well as in inflammation [141]. The PTPN1 gene encodes protein tyrosine phosphatase, non-receptor type 1 that functions as a key regulator of immune homeostasis by inhibiting T-cell receptor signaling and by selectively promoting type I interferon responses after activation of myeloid-cell pattern-recognition receptors [142]. Moreover, the locus between JRKL and CNTN5 also was significantly associated with BD in a study of Spanish population [26]. It is worth noting that these BD-linked loci do not directly reflect the association of the genes, but may be suggestive for further investigation.

## CONCLUSION

Multiple genes have been associated with BD, and they are largely involved in immune activation and regulation. The multigenic feature of BD underlies complex pathogenesis. Some of the reported associations appeared to be conflict in different study cohorts and populations, which suggests the BD-associated polymorphisms of the genes may be ethnic specific, and further verification may be warranted. In addition, some of the BD-associated genes, especially immune regulatory genes, have also been reported in other rheumatic diseases, which supports the shared genetic effects among immune-mediated diseases. Moreover, specific gene polymorphisms were associated with clinical presentation of BD such as ocular lesions, neurological and intestinal involvement. Although functional significance of the BD-associated gene polymorphisms has not been well-defined, understanding the genetics of BD will provide insights into pathogenesis of the disease and an opportunity to interrogate candidate genes in potential diagnostic and therapeutic applications.

## Figures and Tables

**Table 1:** Summary of BD associated genes

**Category**	**Gene**	**Variant**	**Location**	**Associated allele**	**Strongest p value**	**OR**	**Population**	**- **	**Reference**
-	-	-	-	-	-	-	Discovery	Replication	16-33
HLA and related genes	HLA		HLA-B	51	6.8*10-32	20.64	Japanese	Spanish, Chinese, Korean, Italian, Turkish, Ireland, Russia, Germen, Greek, Saudi, Iranian, Jordanian, Tunisian, Israeli	
				15	0.03	0.254	Saudi	Spanish	26,33
				18	0.02	0.58	Spanish	-	26,28
				27	0.003	5.59	Moroccan	-	30
				35	2.46*10-8	0.58	Spanish	-	26,28
				49	2.46*10-8	0.7	Spanish	-	26
				57	1.02*10-5	2.8	Spanish	-	26,28
				58	0.007	0.1	Spanish	-	28
			HLA-A	2	0.003	1.47	Spanish	Korean	26,28,36
				3	0.003	0.53	Spanish	-	26,28
				24	0.01	1.7	Spanish	-	28
				26	0.025	3.87	Spanish	Saudi, Japanese, Korean	26,32,33,34,35,36
				30	0.003	7.53	Korean	Spanish	26,36
				31	0.089	4.06	Spanish	-	26,33
	CIITA	rs12932187	Intron	C	0.01668	0.713	Han Chinese	-	39
	ERAP1	rs10050860	Exon	C	1.1*10-5	0.54	Han Chinese	-	41
		rs17482078 (Arg725Gln)	Exon	C	4.73*10-11	4.56	Turkish	Iranian	40,100
		rs1065407	3’-UTR	A	8.5*10-8	0.51	Han Chinese	-	41
	MICA	-	Exon	MICA*009	0.0009	2.82	Spanish	-	42
				*019	0.0007	6.48	Spanish	-	42
				A5.1	<0.001	0.18	Korean	-	49,50
				A6	6*10-5	2.47	Japanese	Korea, Jordanian, Greek	47,48,49,51
				A9	0.0009	2.82	Japanese	-	51
IL family genes	IL10	rs1518111	Intron	A	1.88*10-8	1.41	Turkish	Iranian, Korean, Japanese, Chinese	56,68,70
		rs1800871	Upstream	T	1.0*10−14	1.45	Japanese	Turkish, Korean, Han Chinese, Western Algeria	55,57,66,69,70
IL family genes	IL10	rs1800872	Upstream	A	2.1*10−14	1.64	Japanese	Turkish, Korean, Han Chinese, Western Algeria	55,57,66,69,70
		rs1554286	Intron	T	3.15*10-5	0.558	Iranian	Korean, Han Chinese	55,66,68,70
	IL12A	rs17810546	Intron	G	1.12*10-10	1.66	Turkish, Mixed population	-	41,61
		rs17810458	Intron	G	1.20*10-5	1.64	Turkish	European	61
	IL23R-IL12RB2	rs1495965	Intergenic	G	1.9*10-11	1.35	Japanese	Turkish, Iranian, Korean	57,68,70
		rs924080	Intergenic	T	2.52*10-4	1.58	Turkish	Japanese, Han Chinese, Korean, Iranian, Western Algeria	56,67-70
		rs11209032	Intergenic	A	3.46 *10-4	1.45	Han Chinese	Western Algeria	67,69
		rs17375018	Intron	G	0.017	1.51	Iranian	-	68
		rs7517847	Intron	T	1.96*10-6	1.48	Iranian	-	68
		rs10489629	Intron	G	0.0017	0.77	Iranian	-	68
		rs1966176	Intron	G	0.0026	1.8	Korean	-	70
	IL23R	rs11209026 (Arg381Gln )	Exon	Gln	8.49*10−9	0.68	Turkish, Japanese	-	65
		rs76418789 (Gly149Arg)	Exon	Arg	0.022	0.54	Turkish, Japanese	-	65
Genes involved in inflammation and autoimmunity	MEFV	rs61752717 (Met694Val)	Exon	Val	1.79*10−12	2.65	Mixed populations	Turkish, Spanish, Greek	65,73-76
		rs28940580 (Met680Ile)	Exon	Ile	0.046	1.74	Turkish	Spanish, Greek	65,74-76
		rs3743930 (Glu148Gln)	Exon	Gln	0.001	3.6	Mixed populations	Turkish, Spanish, Greek	65,73-75
	IRF8	rs17445836	Downstream	G	9.56*10-8	2.044	Han Chinese	-	79
		rs11642873	Downstream	A	9.24*10-7	1.776		-	79
		rs11117433	Intron	C	1.67*10-6	0.63	Turkish	Iranian, Japanese	80
		rs142105922	Intergenic	A	5.58*10-6	0.63			
		rs7203487	Intergenic	C	1.1*10-6	1.38		Iranian	
	TNFAIP3	rs9494885	Intergenic	C	8.35*10-10	1.81	Han Chinese	-	85
		rs10499194	Intergenic	T	0.015	1.96			85
		rs7753873	Intergenic	C	0.015	1.49			85
	REL	rs842647	Intron	G	0.0074	1.57	Chinese	-	90
	TLR4	rs4986790 (Asp299Gl)	Exon	G	8.0*10-12	0.64	Japanese	Turkish, Korean, Chinese	65,93,94
		rs4986791 (Thr399Ile)	Exon	T	8.0*10-12	0.82	Japanese		65,93,94
		rs7037117	3’-UTR	A	0.009	1.64	Japanese	Korean	92-94
	TLR2	rs2289318	Intron	C	6.99*10-6	1.47	Chinese	-	94
		rs3804099	Exon	C	2.43*10-6	0.626	Chinese	-	94
	NOD1	rs2075818	Exon	G	0.0102	0.536	Han Chinese	-	39
Genes involved in inflammation and autoimmunity	NOD2	rs2066844 (Arg702Trp)	Exon	Arg	1.35*10−5	0.4	Caucasians	Japanese, Turkish, Spanish	65,100,102
		rs2066847 (Leu1007fs)	Exon	C	0.0069	0.38	Japanese, Turkish		65,100
		rs2066845 (Gly908Arg)	Exon	Gly	NA	NA	Japanese, Turkish		65
	CCR1 and CC1/CCR3	rs13084057	Intron	G	1.71*10-7	0.32	Han Chinese	-	103
		rs13075270	Intron	C	2.76*10-7	0.32			
		rs13092160	Intron	C	6.50*10-8	0.28			
		rs7616215	Intron	T	5.05*10-19	0.66	Turkish, Japanese	Iranian	40,104
	GIMAP1	rs2286900	Intron	A	9.22*10−6	1.81	Korean, Japanese	-	107
	GIMAP2	rs10266069	Upstream	A	2.57*10−5	1.83	Korean, Japanese	-	
		rs10256482	Upstream	T	2.82*10−5	1.83		-	
	GIMAP4	rs1916012	Upstream	A	2.62*10−2	1.61	Korean, Japanese	-	
		rs1522596	Upstream	A	3.43*10−7	2.38		-	
		rs1608157	Intron	C	6.01*10−8	2.53		-	
	KLRC4	rs2617170	Exon	T	7.98*10-7	0.71	Turkish, Japanese	Iranian	40,104
Genes involved in transcription activation of immune regulation	STAT4	rs7572482	Intron	T	9.77 *10-5	1.68	Han Chinese	-	112
		rs7574070	Intron	T	1.92*10−5	1.27	Turkish	Iranian	40,104,112
		rs897200	Intron	A	5.88 *10-5	1.7	Han Chinese	-	112
	NCOA5	rs2903908	Intron	T	0.016	1.46	Mixed populations	-	115
	FOXP3	rs3761548	Intron	A	0.002	3.841	Iranian	-	118
Others	PSORS1C1	rs12525170	Intron	G	3.01*10-26	3.01	Turkish, Italian	-	124
	FUT2	rs632111	3’-UTR	G	2.09*10-5	1.32	Iranian	Turkish	128
		rs601338	Exon	A	1.18*10-5	1.34			
		rs602662	Exon	A	2.97*10-6	1.35			
		rs492602	Exon	C	4.86*10-5	1.31			
		rs681343	Exon	T	7.27*10-6	1.36			
		rs281377	Exon	C	1.34*10-4	1.3			
	UBAC2	rs9513584	Intron	G	0.0058	1.61	Turkish	Han Chinese	130,132
		rs7999348	Intron	G	0.00058	1.84	Turkish	-	132
		rs3825427	Intron	T	6.9*10-6	1.5	Han Chinese	-	132
		rs9517668	Intron	T	3.3*10-4	1.4	Han Chinese	-	132
		rs9517701	Intron	G	2.9*10-5	1.4	Han Chinese	-	132
		rs9517723	Intron	T	0.0024	1.56	Japanese	-	133
	SUMO4	rs237024	Intron	A	0.0002	1.7	Han Chinese	Tunisian, Korean	136-138
		rs237026	Intron	C	0.03	1.41			136,137
	ADO-EGR2	rs7075773	Intergenic	C	2.96*10−9	0.71	Turkish	-	80
		rs224127	Intergenic	A	1.56*10-6	1.26		Iranian, Japanese	
		rs1509966	Intergenic	A	1.47*10-6	0.8			
	CEBPB-PTPN1	rs913678	Intergenic	C	1.1*10-9	1.33	Turkish	Iranian	80
	JPKL-CNTN5	rs2848479	Intergenic	A	3.29*10-10	1.66	Spanish	-	26
Consider changing to “related genes“									
